# Topical administration of the kappa opioid receptor agonist nalfurafine suppresses corneal neovascularization and inflammation

**DOI:** 10.1038/s41598-021-88118-6

**Published:** 2021-04-21

**Authors:** Hurramhon Shokirova, Takenori Inomata, Tsuyoshi Saitoh, Jun Zhu, Kenta Fujio, Yuichi Okumura, Ai Yanagawa, Keiichi Fujimoto, Jaemyoung Sung, Atsuko Eguchi, Maria Miura, Ken Nagino, Kunihiko Hirosawa, Mizu Kuwahara, Yasutsugu Akasaki, Hiroshi Nagase, Akira Murakami

**Affiliations:** 1 Department of Ophthalmology, Juntendo University Graduate School of Medicine, 3-1-3 Hongo, Bunkyo-ku, Tokyo, 113-0033 Japan; 2 Department of Ophthalmology, Juntendo University Faculty of Medicine, Tokyo, Japan; 3 Department of Strategic Operating Room Management and Improvement, Juntendo University Graduate School of Medicine, Tokyo, Japan; 4 Department of Hospital Administration, Juntendo University Graduate School of Medicine, Tokyo, Japan; 5 Department of Digital Medicine, Juntendo University Graduate School of Medicine, Tokyo, Japan; 6 International Institute for Integrative Sleep Medicine (WPI-IIIS), University of Tsukuba, 1-1-1 Tennodai, Ibaraki, Japan; 7 Department of Ophthalmology, Subei People’s Hospital Affiliated to Yangzhou University, Yangzhou, Jiangsu Province China

**Keywords:** Corneal diseases, Inflammation

## Abstract

Corneal neovascularization (CNV) causes higher-order aberrations, corneal edema, ocular inflammation, and corneal transplant rejection, thereby decreasing visual acuity. In this study, we investigated the effects of topical administration of the kappa opioid receptor agonist nalfurafine (TRK-820) on CNV. To induce CNV, intrastromal corneal sutures were placed on the corneal stroma of BALB/c mice for 2 weeks. Nalfurafine (0.1 µg/2 μL/eye) was topically administered to the cornea once or twice daily after CNV induction. The CNV score, immune cell infiltration, and mRNA levels of angiogenic and pro-inflammatory factors in neovascularized corneas were evaluated using slit-lamp microscopy, immunohistochemistry, flow cytometry, and polymerase chain reaction. The mRNA expression of the kappa opioid receptor gene *Oprk1* was significantly upregulated following CNV induction. Topical administration of nalfurafine twice daily significantly suppressed CNV and lymphangiogenesis, as well as reduced the mRNA levels of angiogenic and pro-inflammatory factors in the neovascularized corneas. Moreover, nalfurafine administration twice daily reduced the numbers of infiltrating leukocytes, neutrophils, macrophages, and interferon-γ-producing CD4^+^ T cells in the neovascularized corneas. In this study, we demonstrated that topical administration of nalfurafine suppressed local CNV in a mouse model along with the activation of KOR, suggesting that nalfurafine may prevent and control CNV in humans.

## Introduction

Cornea is a five-layer transparent avascular tissue that serves a refractive function and as a mechanical barrier^1^. Therefore, transparency of the cornea is essential to maintain visual function. However, corneal transparency may be undermined by corneal neovascularization (CNV), which is caused by factors such as improper use of contact lens, infectious keratitis, trauma, and corneal transplantation. CNV decreases visual acuity, causing higher-order aberrations, corneal edema, ocular inflammation, and corneal transplant rejection^2^. It is estimated that 1.4 million people have CNV in the United States every year^3,4^. In addition, CNV causes blindness in approximately 7 million people worldwide^5^. To date, there are no eye drops for CNV treatment, and this is an important unmet medical need.

Excessive angiogenesis is induced from the limbus to the center of the cornea by the disruption of the balance between angiogenic and anti-angiogenic factors^2^. Angiogenic factors in the cornea include the secreted peptide vascular endothelial growth factor (VEGF) and its receptors (VEGFRs). VEGF-A and its receptor VEGFR-2 are the main regulators of hemangiogenesis, whereas VEGF-C and VEGF-D, along with their receptor VEGFR-3, are the key regulators of lymphangiogenesis^6,7^. In ophthalmology, the anti-VEGF therapy is now commonly administered as an intravitreal injection, mainly for angiogenesis associated with retinal diseases, such as diabetic retinopathy, retinal vein occlusion, or age-related macular degeneration^8^.

Opioids, alkaloids derived from opium poppy, are primarily used for pain relief mediated by their interactions with opioid receptors^9^. There are three main types of opioid receptors: mu, delta, and kappa opioid receptors (MOR, DOR, KORs)^10^. These receptors differ in the selectivity, affinity, and pharmacological effects of various opioid ligands, and are widely expressed not only in the nerve cells but also in the immune system and other non-neuronal tissues, such as endothelial cells^11^. Recent studies in human umbilical vein endothelial cells have revealed anti-angiogenic effects of the KOR agonists U50, 488H^12^ and nalfurafine (TRK-820)^13–15^, mediated by the suppression of the VEGFR expression^16^. Given that KOR activation alleviates pain, if the inhibitory effect of KOR agonists on angiogenesis is also present after local application to the cornea, effective pain management and suppression of excessive vascularization using eye drops may be possible^17^. Among all KOR agonists, only nalfurafine has been shown to cause neither addiction nor aversion; therefore, it was approved as an antipruritic agent for kidney dialysis patients and patients with chronic liver disease in Japan^17^. Therefore, in this study, we investigated the anti-angiogenic effects of the topically administered KOR agonist nalfurafine in a murine CNV model.

## Results

### Increased mRNA expression levels of KOR and angiogenic, lymphangiogenic, and inflammation factors in the neovascularized cornea

**Figure 1:**
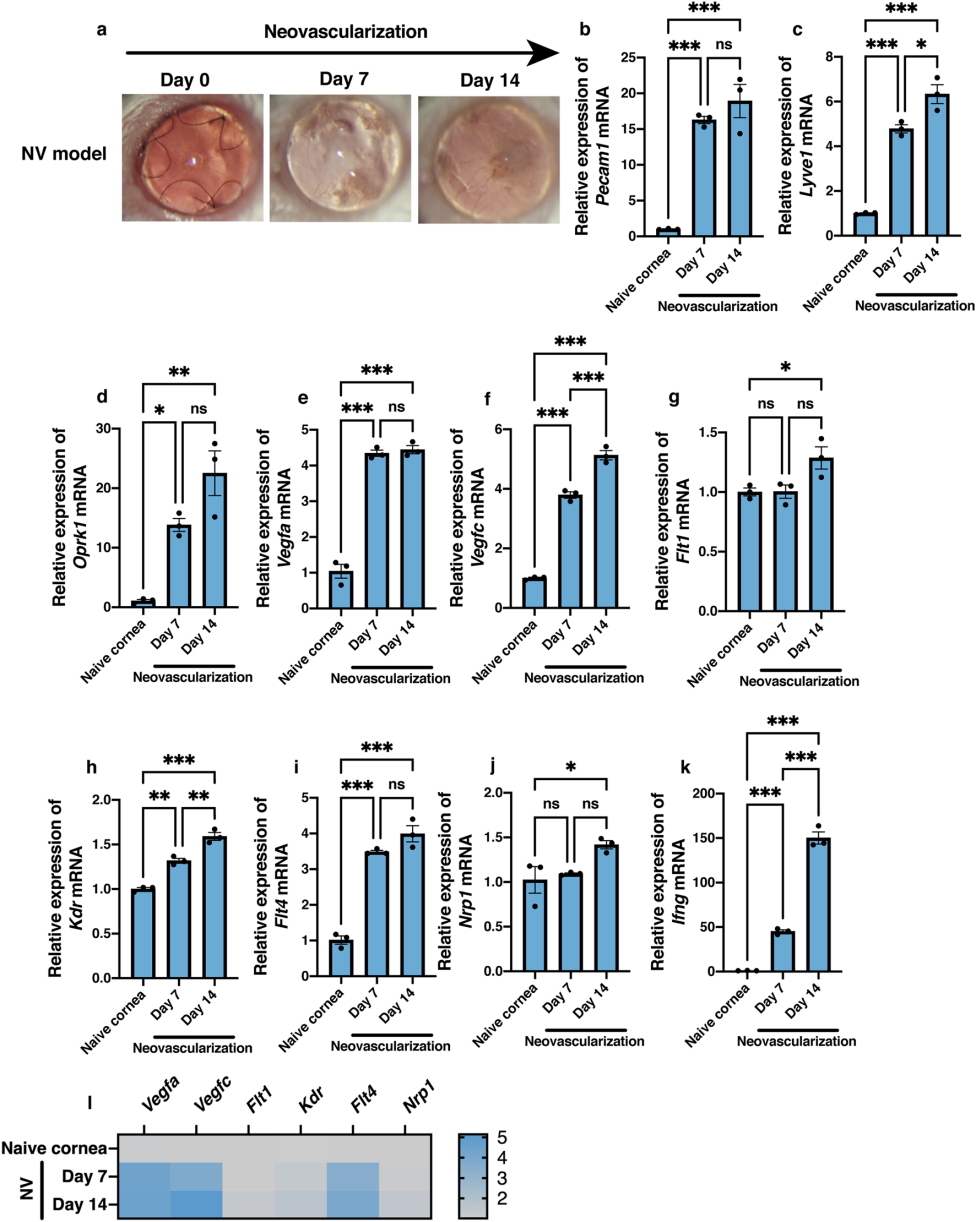
Changes in mRNA expression levels of the angiogenic, lymphangiogenic, and inflammation factors as well as of the kappa opioid receptor in local neovascularized microenvironment. Three intrastromal sutures were placed in the naïve cornea for 14 days. (**a**) Representative slit-lamp images showing corneas post neovascularization (NV) in vivo (magnification × 25). (**b**–**k**) Significant increases in mRNA expression levels were noted at 7 and/or 14 days post neovascularization for *Pecam1* encoding CD31 (**b**), *Lyve1* encoding lymphatic vessel endothelial hyaluronan receptor LYVE1 (**c**), *Oprk1* encoding kappa opioid receptor 1 (**d**), *Vegfa* encoding vascular endothelial growth factor (VEGF) A, (**e**) *Vegfc* (**f**), *Flt1* encoding VEGF receptor 1 (**g**), *Kdr* encoding VEGFR-2 (**h**), *Flt4* encoding VEGFR-3 (**i**), *Nrp1* encoding neuropilin-1 (**j**), and *Ifng* encoding interferon-γ (**k**). Data are presented as the mean ± standard error of the mean (n = 3 in all cases). Statistical significance of differences is illustrated as follows: *P < 0.05; **P < 0.01; ***P < 0.001 (one-way analysis of variance followed by the Bonferroni test). (**l**) Heatmap shows the distribution of fold changes in mRNA levels of angiogenetic and inflammation factors in the cornea after neovascularization induction. ns, no significant difference.

To investigate the effects of CNV induced by suture knots in the central cornea, mRNA levels of angiogenic, lymphangiogenic, and inflammatory factors in the neovascularized tissue were analyzed using reverse transcription-quantitative polymerase chain reaction (RT-qPCR) on days 7 and 14 post neovascularization induction. Figure 1a shows a representative slit photograph of the neovascularized cornea. mRNA levels of *Pecam1* (encoding CD31) and *Lyve1* (encoding lymphatic vessel endothelial hyaluronan) were significantly higher in the cornea affected by neovascularization than in the control group (Fig. 1b, n = 3, *P* < 0.001, one-way analysis of variance [ANOVA] for both days 7 and 14 post-induction; Fig. 1c, n = 3, *P* < 0.001, for both days 7 and 14 post induction). Interestingly, mRNA levels of *Oprk1* that encodes kappa opioid receptors were also significantly increased at days 7 and 14 post neovascularization induction compared to that in the control tissue (Fig. 1d, n = 3, *P* = 0.022 and *P* = 0.002, respectively). *Vegfa* and *Vegfc* mRNA levels were significantly increased at days 7 and 14 post neovascularization induction compared to the level in control (Fig. 1e,f; n = 3, *P* < 0.001). mRNA levels of *Flt1* that encodes VEGFR-1 were significantly increased only at day 14 post neovascularization induction compared to the control level (Fig. 1g, n = 3, *P* = 0.049). In contrast, mRNA levels of *Kdr* and *Flt4* that encode VEGFR-2 and VEGFR-3, respectively, were significantly elevated at days 7 and 14 post neovascularization induction compared to their levels in the control tissue (Fig. 1h,i; n = 3, *Kdr*, *P* = 0.00*1, P* < 0.001, respectively; *Flt4*, *P* < 0.001). In addition, significant elevations of mRNA expression in the cornea affected by neovascularization were noted for *Nrpl*, encoding the VEGF co-receptor neuropilin-1^18^, at day 14 post-induction (Fig. 1j, n = 3, *P* = 0.049) and *Ifng,* encoding the pro-inflammatory cytokine interferon-*γ* (IFN-γ)^19^, at days 7 and 14 post neovascularization induction (Fig. 1k, n = 3, *P* < 0.001). The relative degrees of increases in mRNA expression of several angiogenic factors in the neovascularized tissue are illustrated by a heatmap in Fig. 1l. Particularly high increases in expression levels at days 7 and 14 post-induction compared to the levels in control were noted for the genes encoding angiogenic factors VEGF-A, VEGF-C, and VEGFR-3 (Fig. 1l).

### Downregulation of the mRNA expression of angiogenic factors in the naïve cornea following topical administration of nalfurafine

**Figure 2:**
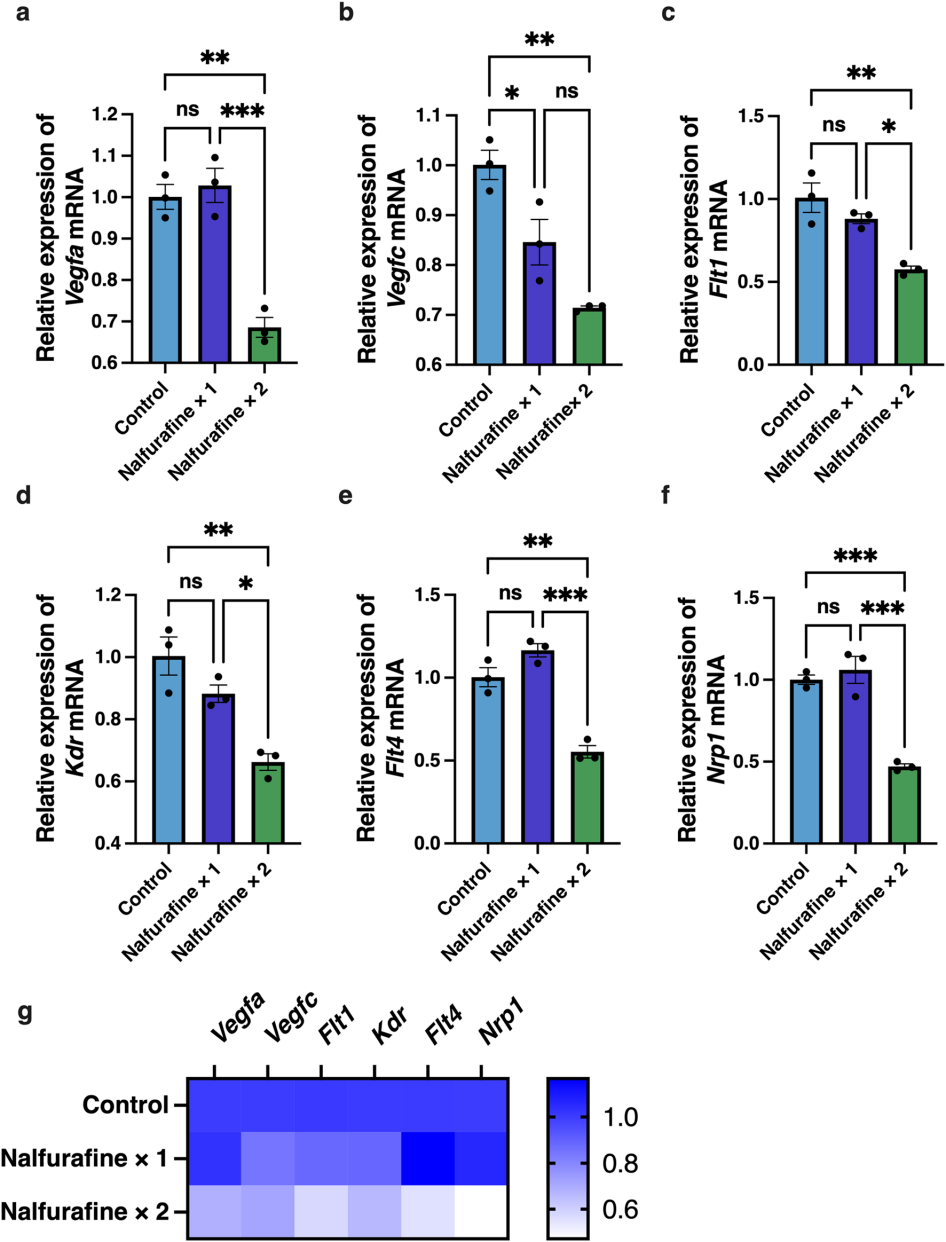
Inhibitory effects of topically administered nalfurafine on the expression of angiogenic factors in the naïve cornea. Eye drops containing the kappa opioid receptor agonist nalfurafine were administered topically (0.1 µg/2 μL/eye) once or twice a day to the naïve cornea of BALB/c mice for 14 days. Relative mRNA expression levels of angiogenic factors in the control and treated naïve corneal samples were analyzed by RT-qPCR. **(a**–**f)** Significant decreases in mRNA levels were noted at 14 days post treatment in samples that were exposed to nalfurafine once and/or twice a day for the following VEGF signaling components: *Vegfa* (**a**), *Vegfc* (**b**), *Flt1* (**c**), *Kdr* (**d**), *Flt4* (**e**), and *Nrp1* (**f**). Data are presented as the mean ± standard error of the mean (n = 3 in all cases). Statistical significance of differences is illustrated as follows: *P < 0.05; **P < 0.01; ***P < 0.001 (one-way analysis of variance followed by the Bonferroni test). (**g**) Heatmap shows relative decreases in expression levels of angiogenic factors in the naïve cornea depending on the dosage of nalfurafine. ns, no significant difference.

To investigate dose-dependent effects of the topically administered KOR agonist nalfurafine on angiogenic signals, we administered it once or twice a day to the naïve cornea. The dosage used (0.1 μg/2 μL/eye) was based on the dosage used in a previous study^16^. The mRNA levels of the genes encoding angiogenic and lymphangiogenic factors were analyzed in the samples of the naïve cornea using RT-qPCR (Fig. 2). We found that *Vegfa*, *Vegfc*, *Flt1*, *Kdr*, *Flt4,* and *Nlp1* mRNA levels were significantly lower in the “nalfurafine twice a day” group than in the control group (Fig. 2a–f, n = 3; *Vegfa*, *P* = 0.001; *Vegfc*, *P* = 0.002; *Flt1*, *P* = 0.004; *Kdr*, *P* = *0.004*; *Flt4, P* = 0.001; and *Nrp1*, *P* < 0.001). Smaller, once a day dosage led to a significant decrease in expression of only *Vegfc* mRNA (Fig. 2b, n = 3, *P* = 0.039). Figure 2g shows a heatmap of relative changes in mRNA expression of the angiogenic factors depending on the dosage.

### Topical administration of nalfurafine upregulated and downregulated the mRNA expression levels of KOR and angiogenic factors, respectively, in neovascularized cornea

**Figure 3:**
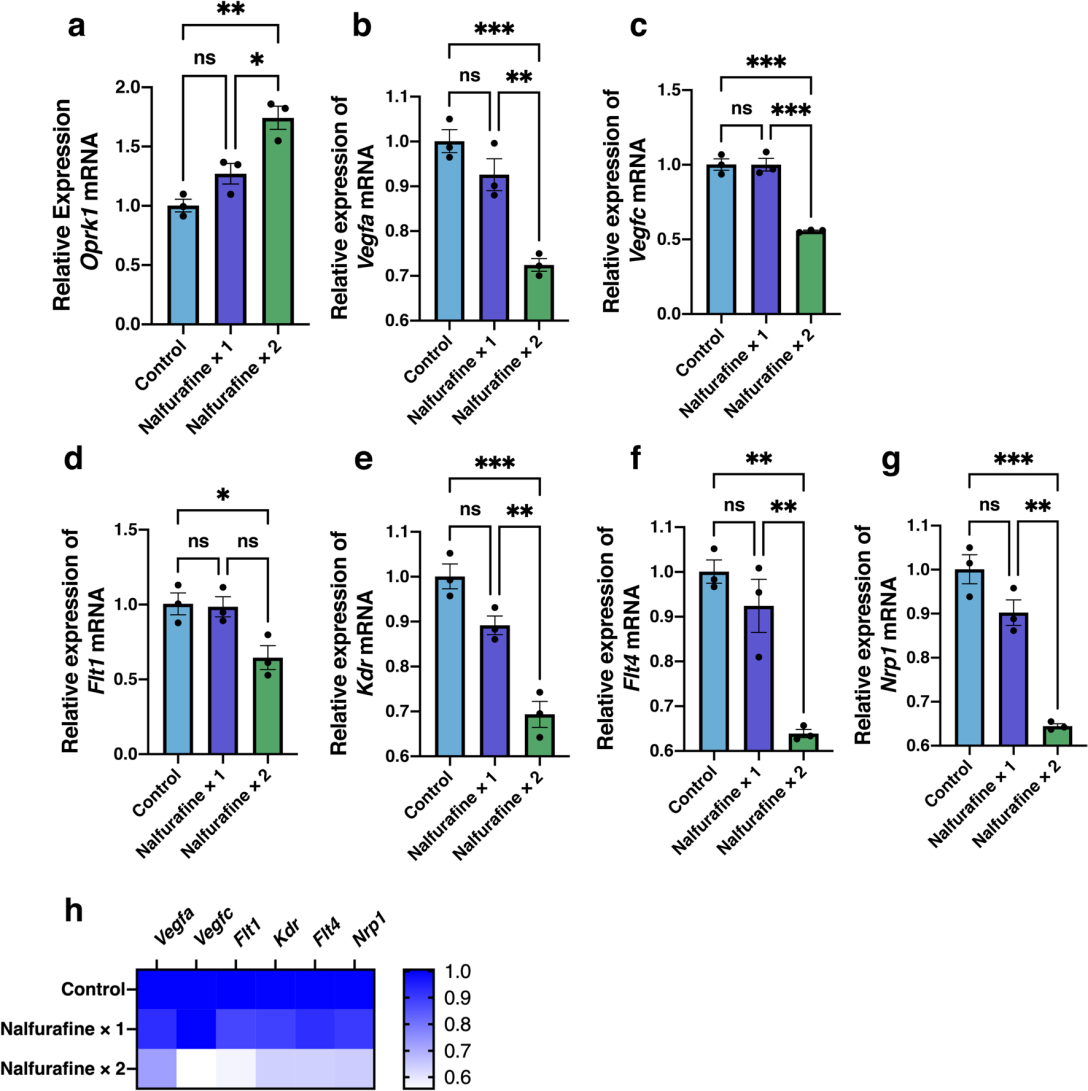
Inhibitory effects of topically administered nalfurafine on the expression of angiogenic factors in the neovascularized cornea. Corneal neovascularization was induced by placing intrastromal figure-of-eight suture knots into the central cornea of BALB/c mice for 14 days. Eye drops containing the kappa opioid receptor agonist nalfurafine were administered topically (0.1 µg/2 μL/eye) once or twice a day to the neovascularized cornea for 14 days. Significant increase in the mRNA expression level of *Oprk1* encoding kappa opioid receptor 1 was observed, 14 days post treatment in the nalfurafine twice a day samples (**a**). Relative mRNA expression levels of angiogenic factors in the control and treated neovascularized corneal samples were analyzed using RT-qPCR (**b**–**g**). Significant decreases in mRNA levels were observed,14 days post treatment in samples that were exposed to nalfurafine once and/or twice a day for the following VEGF signaling components: *Vegfa* (**b**), *Vegfc* (**c**), *Flt1* (**d**), *Kdr* (**e**), *Flt4* (**f**), and *Nrp1* (**g**). Data are presented as the mean ± standard error of the mean (n = 3 in all cases). Statistical significance of differences is illustrated as follows: *P < 0.05; **P < 0.01; ***P < 0.001 (one-way analysis of variance followed by the Bonferroni test). (**h**) Heatmap shows relative decreases in expression levels of angiogenic factors in the neovascularized cornea depending on the dosage of nalfurafine. ns, no significant difference.

Next, we investigated the effects of topical administration of nalfurafine on mRNA expression of KOR and angiogenic and lymphangiogenic factors in the neovascularized cornea (Fig. 3). RT-qPCR results showed that the mRNA levels of *Oprk1* encoding the kappa opioid receptors were significantly upregulated in the nalfurafine twice a day group than in the control group (Fig. 3a, n = 3, *P* = 0.002). We also observed that mRNA levels of *Vegfa*, *Vegfc*, *Flt1*, *Kdr*, *Flt4*, and *Nrp1* were all significantly lower in the nalfurafine twice a day group than in the control group (Fig. 3b–g, n = 3, *Vegfa*, *P* < 0.001; *Vegfc*, *P* < 0.001; *Flt1*, *P* = 0.040; *Kdr*, *P* < 0.001; *Flt4*, *P* = 0.002; and *Nrp1*, *P* < 0.001). Notably, lower dosage (once a day treatment) caused numerical decreases in the expression levels of *Vegfa*, *Kdr*, *Flt4*, and *Nrp1*, but the effect did not reach statistical significance in any of these cases (Fig. 3b–g). Figure 3h shows a heatmap of relative changes in mRNA expression of angiogenic factors in neovascularized cornea depending on nalfurafine dosage. Overall, the expression of angiogenic factors was lower in the nalfurafine twice a day group compared to that in control and nalfurafine once a day groups.

### Topical administration of nalfurafine decreased inflammatory cell infiltration and lowered mRNA expression of inflammation-related molecules in the neovascularized cornea

**Figure 4:**
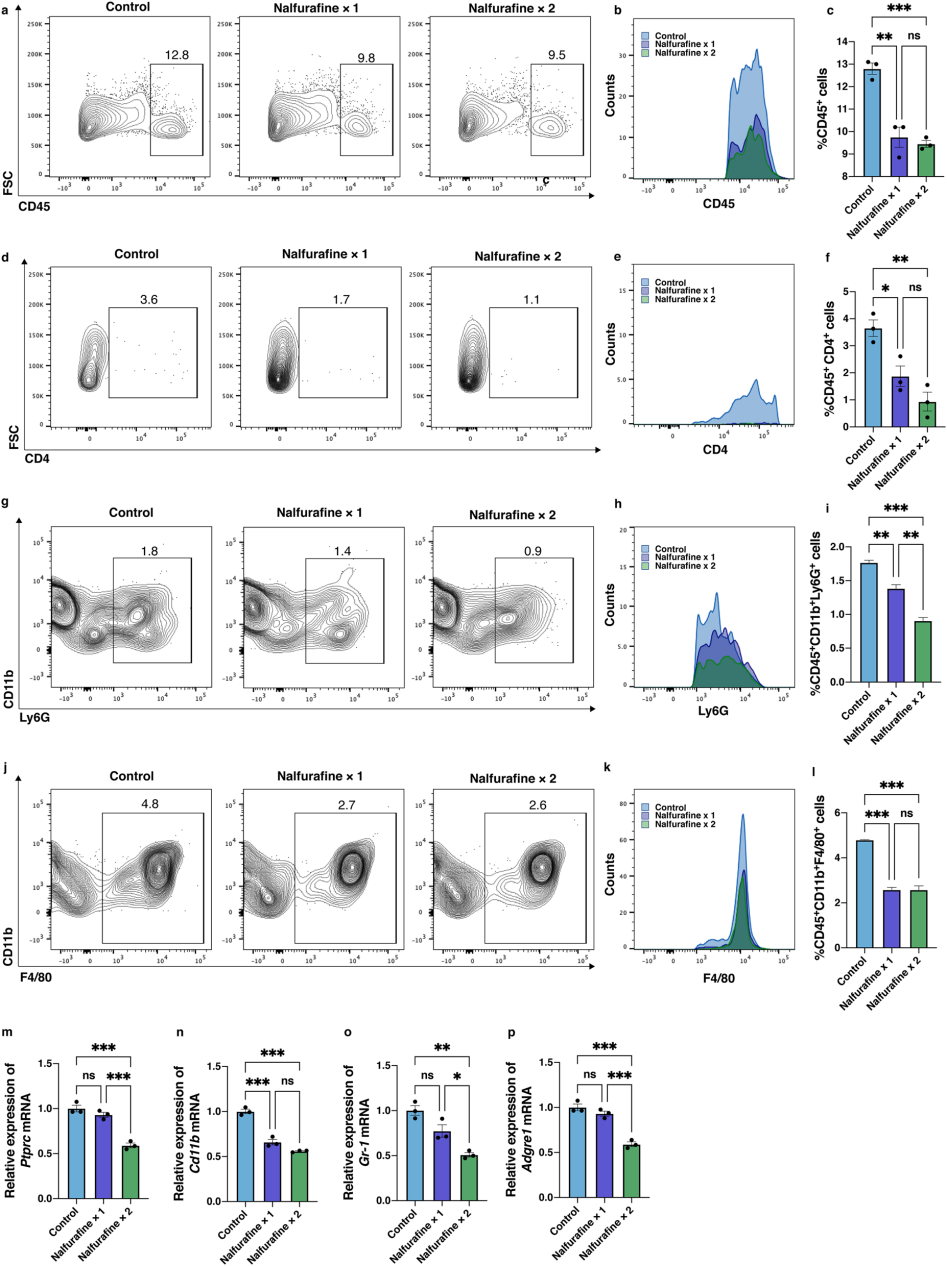
Effects of the kappa opioid receptor agonist nalfurafine on immune cell infiltration in neovascularized cornea. Representative flow cytometry plots (**a**) and the histogram (**b**) showing the frequency of CD45^+^ leukocytes. (**c**) Statistical analyses of flow cytometry data showing the numbers of CD45^+^CD4^+^ infiltrating T cells in the neovascularized corneas 14 days post neovascularization induction in control conditions and following treatment with nalfurafine (0.1 µg/2 μL/eye) once or twice a day. Representative flow cytometry plots (**d**) and the histogram (**e**) showing the frequency of CD45^+^CD4^+^ infiltrating T cells in the neovascularized corneas 14 days post neovascularization induction in control conditions and following treatment with nalfurafine (0.1 µg/2 μL/eye) once or twice a day. (**f)** Statistical analyses of flow cytometry data showing the numbers of CD45^+^CD4^+^ infiltrating T cells. Representative flow cytometry plots (Fig. 4 g) and the histogram (Fig. 4 h) showing the frequency of CD45^+^CD11b^+^Ly6G^+^ neutrophils in the neovascularized corneas 14 days post neovascularization induction in control conditions and following treatment with nalfurafine (0.1 µg/2 μL/eye) once or twice a day. The number of CD45^+^CD11b^+^Ly6G^+^ neutrophils was significantly lower in tissues treated with topical administration of nalfurafine once or twice a day compared with the control group (Fig. 4i, n = 3, *P* = 0.004, *P* < 0.001, vs. control, respectively). Representative flow cytometry plots (Fig. 4j) and the histogram (Fig. 4 k) showing the frequency of CD45^+^CD11b^+^F4/80^+^ macrophage. The abundance of CD45^+^CD11b^+^F4/80^+^ macrophage infiltrating the corneas was significantly reduced in the corneal samples treated with nalfurafine once or twice a day (Fig. 4 l, n = 3, *P* < 0.001, *P* < 0.001, vs. control, respectively). mRNA levels of *Ptprc* (Fig. 4 m), *CD11b* (Fig. 4n), *Gr-1* (Fig. 4o), and *Adgre1* (Fig. 4p) were significantly reduced following topical administration of nalfurafine twice a day, 14 days post neovascularization induction.

Infiltrating inflammatory cells secrete VEGF and inflammatory cytokines^20–24^. To determine the effect of nalfurafine on inflammatory cell infiltration in the neovascularized cornea, the numbers of CD45^+^ leukocytes, CD4^+^ T cells, CD45^+^CD11b^+^Ly6G^+^ neutrophils, and CD45^+^CD11b^+^F4/80^+^ macrophages infiltrating the neovascularized cornea were assessed by using flow cytometry (Fig. 4a–l). Flow cytometry plots (Fig. 4a) and the histogram (Fig. 4b) illustrate the number of CD45^+^ leukocytes. The number of CD45^+^ cells was significantly lower in the tissues treated by topical administration of nalfurafine once or twice a day compared to that in the control group (Fig. 4c, n = 3, *P* = 0.001, *P* < 0.001, respectively). Flow cytometry plots (Fig. 4d) and the histogram (Fig. 4e) illustrate the frequency of CD4^+^CD45^+^ T cells. The abundance of CD4^+^CD45^+^ T cells infiltrating the corneas was also significantly reduced in the corneal samples that were treated by nalfurafine once or twice a day (Fig. 4f, n = 3, *P* = 0.030, *P* = 0.004, respectively). Flow cytometry plots (Fig. 4g) and the histogram (Fig. 4h) illustrate the frequency of CD45^+^CD11b^+^Ly6G^+^ neutrophils. The number of CD45^+^CD11b^+^Ly6G^+^ neutrophils was significantly lower in tissues treated with topical nalfurafine once or twice a day compared with the control group (Fig. 4i, n = 3, *P* = 0.004, *P* < 0.001, respectively). Flow cytometry plots (Fig. 4j) and the histogram (Fig. 4k) illustrate the frequency of CD45^+^CD11b^+^F4/80^+^ macrophage. The abundance of CD45^+^CD11b^+^ F4/80^+^ macrophages infiltrating the corneas was also significantly reduced in samples treated with nalfurafine once or twice a day (Fig. 4l, n = 3, *P* < 0.001, *P* < 0.001, respectively). Decreased mRNA expressions of *Ptprc* (CD45, Fig. 4m, n = 3, *P* < 0.001), *Cd11b* (Fig. 4n, n = 3, *P* < 0.001, *P* < 0.001, respectively), *Gr-1* (Ly6G. Fig, 4**0**, n = 3, *P* = 0.002), and *Adgre1* (F4/80*,* Fig. 4p, *P* < 0.001) were observed in the corneal samples treated with nalfurafine twice a day.

**Figure 5:**
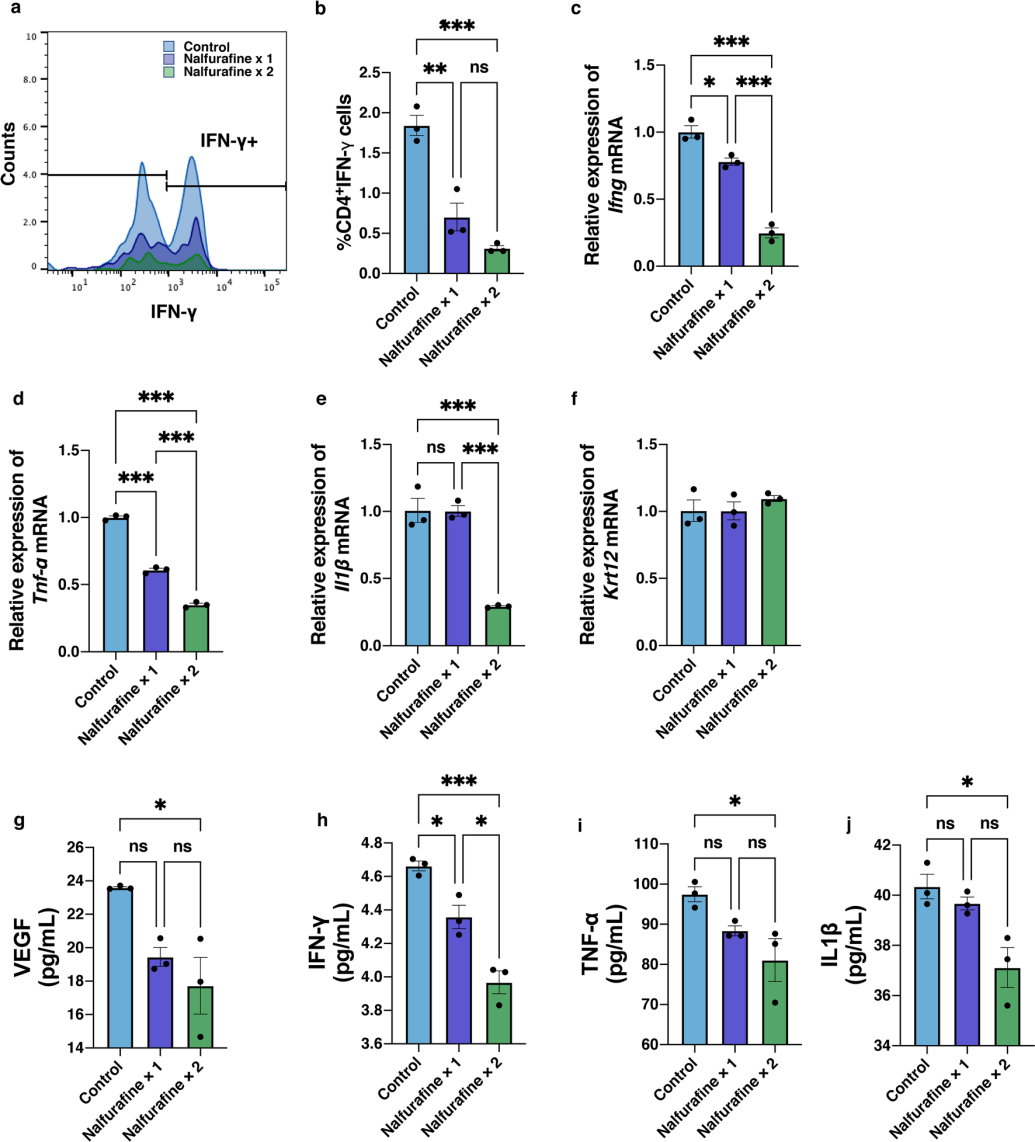
Effects of the kappa opioid receptor agonist nalfurafine on IFN-γ^+^CD4^+^ T cells and expression of inflammation-related cytokines. (**a**) Flow cytometry analysis of IFN-γ expression in CD4^+^ T cells. (**b**) Statistical analysis of flow cytometry data showing the numbers of IFN-γ^+^CD4^+^ T cells in neovascularized corneas at day 14 after neovascularization induction. (**c**) mRNA levels of *Ifng,* encoding interferon-γ, were significantly reduced by topical administration of nalfurafine once and twice at day 14 post neovascularization induction. (**d**) mRNA levels of *Tnf-α* were significantly reduced following topical administration of nalfurafine twice a day, 14 days post neovascularization induction. (**e)** mRNA levels of *Il1β* were significantly reduced following topical administration of nalfurafine once and twice a day, 14 days post neovascularization induction. (**f**) mRNA levels of *Krt12*, encoding cytokeratin 12, were not significantly altered by the application of nalfurafine. The protein levels of VEGF (**g**), IFN-γ (h), TNF-*α* (**i**)*,* and IL1*β* (**j**) measured using ELISA were significantly reduced following topical administration of nalfurafine twice a day, 14 days post neovascularization induction. Data are presented as the mean ± standard error of the mean (n = 3 in all cases). Statistical significance of differences is illustrated as follows: ns, no significant difference; *P < 0.05; **P < 0.01; ***P < 0.001 (one-way analysis of variance followed by the post hoc Bonferroni test). IFN, interferon; Tnf-α, Tumor necrosis factor-α; Il1β, interleukinβ; ELISA, enzyme-linked immunosorbent assay.

Figure 5 shows the expressions of CD4^+^IFN-γ^+^ T cells and inflammation cytokines in the neovascularized cornea following topical administration of nalfurafine. IFN-γ-producing activated T cells are recognized as the principal mediators of corneal inflammatory responses^25,26^. The numbers of CD4^+^IFN-γ^+^ T cells in the neovascularized cornea were analyzed at day 14 post neovascularization induction (Fig. 5a). Flow cytometry analysis showed a marked reduction in the quantity of CD4^+^IFN-γ^+^ T cells in the neovascularized corneal samples treated with topically administered nalfurafine once or twice a day compared to their number in the control group (Fig. 5b, n = 3, *P* = 0.002, *P* < 0.001, respectively). Additionally, the *Ifng* mRNA level was significantly decreased in the neovascularized cornea after the treatment with topically administered nalfurafine once or twice a day compared to that in control samples (Fig. 5c, n = 3, *P* = 0.017, *P* < 0.001, respectively). Among the mRNA expressions of inflammatory cytokines, *Tnf-α* was decreased following nalfurafine once or twice a day treatment (Fig. 5d, n = 3, *P* < 0.001, *P* < 0.001, respectively), while *Il1β* was suppressed only in the twice a day group (Fig. 5e, n = 3, *P* < 0.001). Moreover, mRNA expression levels of *Krt12* that encodes the corneal epithelial differentiation marker cytokeratin 12^27^ did not differ significantly between the groups (Fig. 5f, n = 3, *P* > 0.05).

The protein levels of VEGF, IFN-γ, TNF-α, and IL1β were measured using enzyme-linked immunosorbent assay (ELISA). VEGF (Fig. 5g, *P* = 0.018, *P* = 0.006, respectively ), IFN-γ (Fig. 5h, P = 0.032, *P* < 0.001, respectively), TNF-*α* (Fig. 5i, *P* = 0.040)*,* and IL1β (Fig. 5j, *P* = 0.020) expression levels were significantly reduced in the nalfurafine once or twice a day treatment compared with the control group.

### Inhibition of corneal angiogenesis and lymphangiogenesis by topical administration of nalfurafine

**Figure 6:**
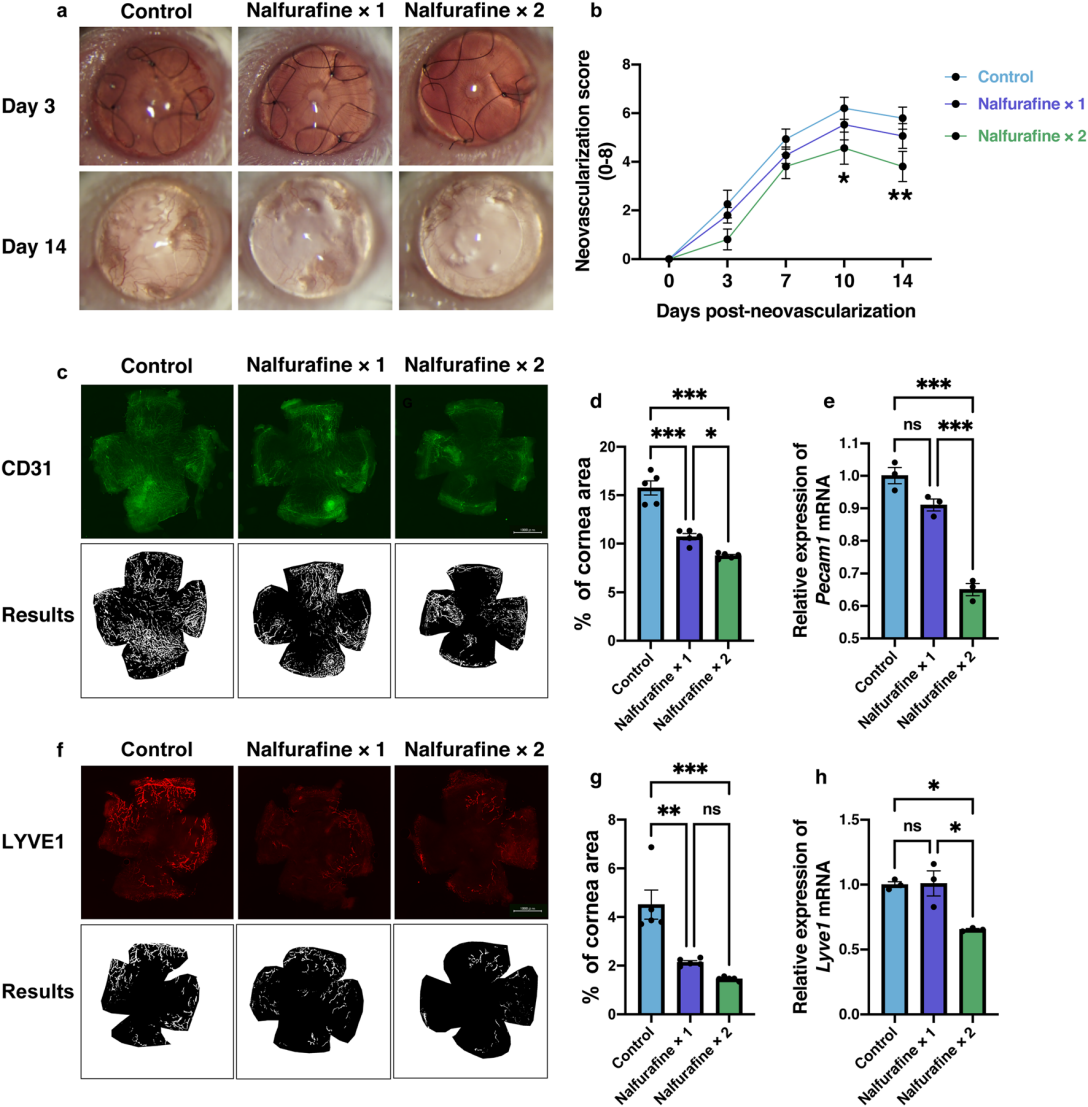
Modulation by the kappa opioid receptor agonist nalfurafine of the hemangiogenesis and lymphangiogenesis in neovascularized cornea. (**a**) Slit-lamp images showing corneas at 3 and 14 days after neovascularization induction in vivo (magnification × 25) (**b**) Neovascularization scores in the corneas 14 days after neovascularization induction (mean ± standard error of the mean, n = 15 per group; statistical significance is indicated as follows: *P = 0.034, **P = 0.007; two-way ANOVA followed by the post hoc Bonferroni test). (**c**) Representative images showing CD31 staining of the whole cornea and the processed images. (**d**) The fractions of the total cornea area covered with blood vessels (CD31^+^) were significantly reduced in corneas that were treated with nalfurafine once or twice a day compared to that in control, untreated corneas on day 14 post neovascularization induction (mean ± standard error of the mean, n = 5, statistical significance is indicated as follows: *P = 0.033 and ***P < 0.001 vs. control). (**e**) Relative mRNA expression levels of *Pecam1*, encoding CD31, were significantly decreased in corneas that were treated with nalfurafine twice a day compared to that in control, untreated corneas on day 14 post neovascularization induction (mean ± standard error of the mean, n = 3, statistical significance is indicated as follows: ***P ≤ 0.001 vs. control). (**f**) Images showing lymphatic vessel endothelial hyaluronan receptor1 (LYVE1) staining of the whole cornea and the processed images. (**g**) The fractions of the cornea area covered with lymphatic vessels were significantly reduced in corneas that were treated with nalfurafine once or twice a day compared with the control, untreated corneas on day 14 post neovascularization induction (mean ± standard error of the mean, n = 3, **P < 0.01, ***P < 0.001 vs. control, respectively). (**h**) Relative *Lyve1* mRNA expression was significantly decreased in corneas that were treated with nalfurafine twice a day compared to that in control, untreated corneas on day 14 post neovascularization induction (mean ± standard error of the mean, n = 3, statistical significance is indicated as follows: *P = 0.016, vs. control). ns no significant difference.

Figure 6a shows representative images of neovascularized cornea at days 3 and 14 after neovascularization induction following topical administration of nalfurafine. Topical administration of nalfurafine twice a day significantly reduced the neovascularization score compared to that in the control group at days 10 and 14 post neovascularization induction (Fig. 6b, n = 15 per each group; *P* = 0.034, *P* = 0.007, respectively, two-way ANOVA followed by the post hoc Bonferroni test). Figure 6c shows representative images of CD31 staining of the whole cornea and the processed images on day 14 post neovascularization induction. Fractions of the corneal area covered with blood vessels (CD31^+^) in the samples treated with topically administered nalfurafine once or twice a day were significantly smaller than those in the control group (Fig. 6d, n = 5, *P* < 0.001). Furthermore, the mRNA level of *Pecam1* was significantly reduced by the treatment with nalfurafine twice a day compared to the level in the control group on day 14 post neovascularization induction (Fig. 6e, n = 3, *P* < 0.001). Figure 6f shows representative images of LYVE1 staining of the whole cornea and its processed images on day 14 post neovascularization induction. The fractions of the corneal area covered with lymphatic vessels (LYVE1^+^) in the samples exposed to topically administered nalfurafine once or twice a day were significantly reduced compared to that in the control group (Fig. 6g, n = 5, *P* < 0.01, *P* < 0.001). In addition, *Lyve1* mRNA level was significantly decreased in corneal samples after topical administration of nalfurafine twice a day on day 14 post neovascularization induction (Fig. 6h, n = 3, *P* = 0.016).

## Discussion

Effective treatment of CNV represents an unmet medical need and is critical for the retention of visual function. In this study, we investigated the efficacy of the topical administration of the KOR agonist nalfurafine in the treatment of CNV in mice. We demonstrated that topical administration of nalfurafine attenuated angiogenesis and immunological responses by upregulating KOR expression levels and downregulating expression levels of several angiogenic and inflammatory factors in the cornea. These results suggest that topical administration of nalfurafine and other KOR agonists might be an effective treatment option for the management of CNV.

**Figure 7:**
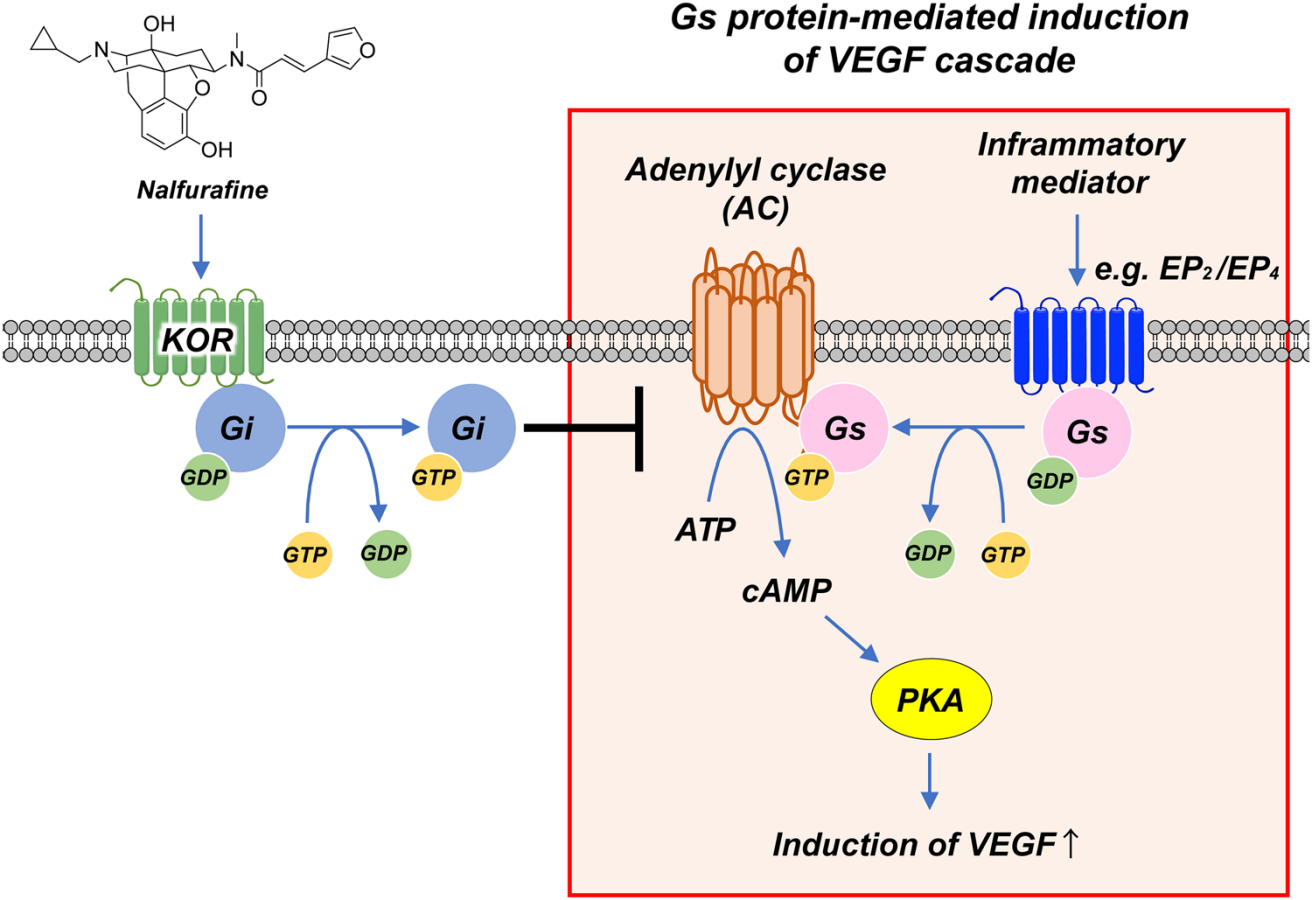
Mechanism of the effect of the kappa opioid receptor agonist on local neovascularization. Kappa opioid receptor (KOR) agonist transduces signals through the Gi protein to inhibit adenylyl cyclase (AC) and subsequently decrease cyclic adenosine monophosphate (cAMP) production and inactivate protein kinase A (PKA). EP, prostaglandin E receptor; GDP, guanosine diphosphate; GTP, guanosine triphosphate; VEGF, vascular endothelial growth factor.

We showed that CNV induction in the cornea led to a significant increase in the expression of *Oprk1* that encodes KOR (Fig. 1d), confirming the notion about the stimulatory effect of peripheral inflammation on the expression of KOR in the corneal microenvironment in accordance with a previous study^28^. Another previous study showed the increased expression of KOR in human umbilical vein endothelial cells in vitro^16^. These results indicate that the increased expression of KOR may be induced by the increased vascular endothelial cells itself in the local cornea during the angiogenesis process. As shown by the schematic in Fig. 7, inflammatory mediators activate G proteins that upregulate cyclic adenosine monophosphate (cAMP) generation, which in turn activates the protein kinase A pathway for VEGF production, whereas KOR activation suppresses VEGF production by promoting Gαi/o inhibition of adenylyl cyclase, which decreases cAMP levels and attenuates protein kinase A activation^29,30^ It has been reported that the downregulation of KOR expression in hepatocellular carcinoma had a strong association with poor prognosis^31^. We showed here that topically administered nalfurafine, which activates KOR, inhibited the expression of genes encoding VEGF-A, VEGF-C, as well as VEGFR-1–3 (Figs. 2, 3), resulting in decreased CNV and lymphangiogenesis in neovascularized corneas (Fig. 6). These findings suggest that the KOR system controls the development of blood and lymphatic vessels in the corneal microenvironment^32^. Therefore, ligands that activate the KOR system, such as nalfurafine, may be useful as anti-angiogenic agents in the cornea.

CNV occurs as a result of an imbalance between angiogenic and anti-angiogenic factors in favor of angiogenic molecules^32–35^. This imbalance causes pathogenic angiogenesis in CNV, which in turn activates pro-angiogenic factors including VEGF, VEGFR, IFN-γ, TNF-α, and IL1β in the neovascularized microenvironment and strongly potentiates further development of CNV^21,36,37^. Therefore, controlling the expression levels of VEGF, VEGFR, and inflammation cytokines is an effective strategy to inhibit angiogenesis in the local corneal tissue^21,38,39^. In this study, topical administration of nalfurafine twice a day significantly inhibited CNV compared to the extent of the pathology in the untreated control or nalfurafine once a day groups (Fig. 5). In addition, as shown in Fig. 3, *Vegfa* mRNA expression was significantly reduced in the treatment group administered nalfurafine twice a day compared to that in the treatment group administered nalfurafine once a day or in the untreated group. This result suggests that sustained KOR activation achieved by topical administration of nalfurafine inhibits CNV. Furthermore, we showed that treatment with nalfurafine downregulated the expression of genes that encode the VEGF co-receptors VEGFR-2 and neuropilin-1^18^ in the neovascularized cornea. A previous study has reported that KOR activation suppresses the expression of VEGFR-2 and neuropilin-1 by inhibiting cAMP/PKA signalling^40^. Furthermore, because lymphatic vessels multiply during angiogenesis^41^, the decreased expression of VEGFR-3 is secondary to the suppression of increased angiogenesis by KOR activation, which also suppresses lymphangiogenesis. Here, we found that topical administration of nalfurafine to the neovascularized cornea reduced the expression of genes encoding VEGF and VEGF-R, which is in agreement with previous studies^16,42^, further confirming that nalfurafine may be effective as a topical agent in CNV.

In CNV, angiogenesis of the corneal local tissue is accompanied by the induction of local inflammation, which damages the corneal epithelium and stroma, leading to the infiltration of inflammatory cells, primarily neutrophils and macrophages^6,8,43–45^. Infiltrating neutrophils secrete VEGF^20,21^. Abnormally growing blood and lymphatic vessels trigger local infiltration of inflammatory cells to the cornea and further promote angiogenesis^38,46^. Topical administration of nalfurafine reduced the number of infiltrating CD45^+^CD4^+^ and CD4^+^ IFN-γ^+^ cells, CD45^+^CD11b^+^Ly6G^+^ neutrophils, CD45^+^CD11b^+^F4/80^+^ macrophage, and inflammatory cytokine expression in the neovascularized cornea in our experiments (Figs. 4, 5). Because nalfurafine inhibited the infiltration of these cells, the secretion of VEGF-A and VEGF-C may have been reduced. This indicates that activating the KOR system suppressed the infiltration of inflammatory cells to the damaged sites in the neovascularized cornea likely by reducing the levels of associated inflammatory cytokines.

In infectious keratitis and corneal transplantation concurrent with CNV, both the suppression of angiogenesis and pain control are important. Opioid systems are mainly responsible for the control of neurophysiological functions, including pain, mood changes, physical dependence, reward, and addictive behaviours^47^. The opioid system comprises MOR, DOR, and KORs and their endogenous ligands^48^. The KOR agonists are considered as promising alternatives to the MOR agonists: the former also have strong analgesic properties but are devoid of the side effects caused by MOR activation. Given that we showed the suppressive effects of the KOR agonist nalfurafine on CNV in mice, it is possible that eye drops containing nalfurafine may be able to control pain as well as suppress angiogenesis in local corneal tissue.

This study has several limitations. We identified the inhibitory effects of nalfurafine on CNV in a murine model. However, because CNV may be triggered by various pathological conditions, further studies using alternative CNV models, such as the alkali burn model and corneal micropocket assay should be considered. Although sustained activation of KOR with a selective agonist was thought to be important for the inhibition of CNV, the optimal concentration and doses of nalfurafine for topical administration to the cornea were not determined in this study because we used the previously published dosing regimen for this drug^16^. In addition, the treatment with much higher doses of KOR agonists for longer periods might lead to the development of tolerance to kappa opioids, and this possibility should be investigated^16,49^. Furthermore, although a potential advantage of using nalfurafine and other KOR agonists is the combination of attenuated angiogenesis and pain relief due to their analgesic effects, we did not directly investigate pain in this study. Moreover, we did not compare the anti-angiogenic effects of nalfurafine with those of other KOR agonists^50^. Therefore, further studies are required to assess the possible anti-angiogenic effects of a broader spectrum of KOR agonists in CNV. Nonetheless, our study is an important step forward, as there is currently no ophthalmic medication that both provides pain relief and has anti-angiogenic effects in clinical ophthalmic practice. The results of our study warrant further experiments to clarify the effects of nalfurafine and other KOR agonists and pave way to the potential clinical application of these drugs for CNV treatment.

In conclusion, we have shown that that topical administration of nalfurafine, a clinically used KOR agonist, suppressed local CNV in a mouse model. Therefore, KOR agonists might be useful for preventing and controlling CNV.

## Methods

### Animals

Six- to eight-week-old BALB/c (H-2d) male mice were purchased from Sankyo Labo Service Corporation, Inc. (Tokyo, Japan). All animal experiments were approved by the Institutional Animal Care and Use Committee of the Juntendo University Graduate School of Medicine (Approval No. 1271) and were conducted in compliance with the Association for Research in Vision and Ophthalmology statement for the Use of Animals in Ophthalmic and Vision Research and the ARRIVE guidelines.

### Topical administration of eye drops containing nalfurafine

The KOR agonist (-)-17-cyclopropylmethyl-3,14b-dihydroxy-4,5a-epoxy-6b-[N-methyl-trans-3-(3-furyl) acrylamido] morphinan hydrochloride (nalfurafine, TRK-820, TORAY, Tokyo, Japan)^13,14^ has been approved in Japan as a treatment for hemodialysis-related uremic pruritus^17^. Nalfurafine was topically administered to the naïve or neovascularized cornea once or twice a day (0.1 µg/2 μL/eye). The dose-dependent effects of topically administered nalfurafine on angiogenic signals were investigated by once a day or twice a day application of the drug to the naïve cornea.

### Suture-induced CNV model

A murine CNV model was created as previously described^41,51^. First, 1.5-mm trephine was used to mark the central cornea of BALB/c mice. Three intrastromal figure-of-eight suture knots were placed into the central cornea using 11–0 nylon sutures (AB-0550S; MANI, Tochigi, Japan) for 14 days.

### Assessment of cornea neovascularization

CNV was evaluated once a week using a slit-lamp biomicroscope for 8 weeks. We used the standard neovascularization grading scheme (range from 0 to 8)^52^. Neovascularization grading were assigned as follows: 0, no vessels; 1, vessels peripheral cornea only (1–2 quadrants); 2, vessels peripheral cornea only (3–4 quadrants); 3, vessels marked central corneal border (1–2 quadrants), 4, vessels marked central corneal border (3–4 quadrants); 5, vessels peripheral marked central cornea (1–2 quadrants); 6, vessels peripheral marked central cornea (3–4 quadrants); 7, vessels central marked central cornea (1–2 quadrants); 8, vessels central marked central cornea (3–4 quadrants).

### RNA isolation and RT-qPCR

The excised corneas were immediately submerged in the RNAlater solution (Ambion, Austin, TX, USA). Total RNA was isolated from five corneas per group by using a NucleoSpin RNA isolation kit (Macherey-Nagal GmbH, Duren, Germany). The cDNA was reverse-transcribed from total RNA by using random primers and a ReverTra Ace qPCR RT kit (Toyobo, Osaka, Japan) following the manufacturer’s guidelines. The qPCR primers specific for mouse mRNA are shown in Table 1. qPCR was performed with the Applied Biosystems 7500 Fast Real-Time PCR Systems using a FAST-SYBR Green master mix (Thermo Fisher Scientific K.K., Tokyo, Japan). Results were analyzed using the comparative cycle threshold method, and *Gapdh* mRNA expression in the same cDNA sample was used as the internal control.

**Table 1:** Primer sets for real-time PCR.

Gene	Forward (5′–3′)	Reverse (5′–3′)
*Adgre1*	TCTGGGGAGCTTACGATGGA	TGGTTCTGAACAGCACGACA
*Cd11b*	CGGAAGGATTCAGCAAGCCAGAAC	AGCTGGACTCAGCAGGCTTTAC
*Flt1*	GAGGAGGATGAGGGTGTCTATAGGT	GTGATCAGCTCCAGGTTTGACTT
*Flt4*	GTGCTCAAAGAGGTGACCGA	TGAGGAGGCACATTCACCAC
*Gapdh*	AAGGGCTCATGACCACAGTC	GGATGACCTTGCCCACAG
*Gr-1*	GGGAGGGGCTGAGAGAAAGTA	AGGGCTGCACAGATAAAACTTCC
*Ifng*	CGGCACAGTCATTGAAAGCC	TGTCACCATCCTTTTGCCAGT
*Il1β*	TGCCACCTTTTGACAGTGATG	ATGTGCTGCTGCGAGATTTG
*Kdr*	GCCCTGCCTGTGGTCTCACTAC	CAAAGCATTGCCCATTCGAT
*Krt12*	CGCTGGGTCTCAGAGTGATT	CTGACTCTGGCAGAAACGATCTTA
*Lyve1*	TGGGAAGAATGGCAAAGGTGTCC	ATGCAGGAGTTAACCCAGGTGTCG
*Nrp1*	TGTGAAGTGGAAGCCCCTAC	CACCTGTGAGCTGGAAGTCA
*Oprk1*	AGTCCCCCATTCAGATCTTCC	ACAGCAATGTAGCGGTCCAC
*Pecam1*	TTGAGCCTCACCAAGAGAACGG	ACTCTCGCAATCCAGGAATCGG
*Ptprc*	GAGCACAACAGAGAATGCCC	AGCGTGGATAACACACCTGG
*Tnf-α*	AGCCCACGTCGTAGCAAAC	TTTGAGATCCATGCCGTTGG
*Vegfa*	TGTACCTCCACCATGCCAAGTG	TGGGACTTCTGCTCTCCTTCTGTC
*Vegfc*	TCCTGGGAAATGTGCCTGTGAATG	GCACACGGTCTTCTGTAACAACTG

### Flow cytometry analysis

Corneas were harvested, and single-cell suspensions were prepared as described previously^52,53^. To avoid non-specific staining, cells were blocked with an anti-FcR blocking antibody (eBioscience, San Diego, CA, USA). Isolated cells were stained with the respective antibodies. For intracellular IFN-γ staining, cells were stimulated with 50 ng/mL phorbol-12-myristate-13-acetate and 500 ng/mL inomycin (Sigma-Aldrich, St. Louis, MO, USA) for 6 h at 37 °C in the atmosphere of 95% air and 5% CO_2_ in the presence of GolgiStop (0.7 μL per 100 μL cell culture; BD Biosciences, San Jose, CA) to inhibit cytokine secretion. The cells were then stained with the following antibodies: anti-CD4 (GK1.5), anti-CD45 (30-F11), anti-CD11b (M1/70), anti-Ly6G (1A8), anti-F4/80 (BM8), and anti-IFN-γ (XMG1.2) (Biolegend, San Diego, CA, USA). All antibodies, their matched isotype controls as well as fixation and permeabilization buffers were purchased from eBioscience. Stained cells were examined by using an LSR Fortessa cell analyzer (BD Biosciences, Franklin Lakes, NJ, USA) and analyzed by using FlowJo software X 10.5.3. (FlowJo LLC, Ashland, OR, USA; purchased from https://www.flowjo.com).

### Enzyme-linked immunosorbent assay

Neovascularized murine corneas were enucleated and homogenized using a Micro Smash MS-100 homogenizer (Tomy Seiko co., LTD, Japan), for 30 s power at 3,500 rpm, two times, and centrifuged at 5,000 × *g*, 5 min to remove tissue debris. Supernatants were collected and quantified for ELISA according to the manufacturer’s instructions using ELISA kits for mouse VEGF (DY493-05), IFN-γ (DY485), TNF-α (DY410-05), and IL1β (DY401-05) (R&D Systems Minneapolis, MN, USA).

### Corneal whole mount and immunofluorescence staining

Corneas were excised on day 14 after suture-induced CNV and washed with phosphate-buffered saline. The corneal epithelium was removed after incubation with 20 mM EDTA for 60 min at 37 °C, fixed in acetone for 15 min at 20–22 °C, and blocked in 2% bovine serum albumin for 60 min. Corneas were then double-stained for CD31 and lymphatic vessel endothelial hyaluronan receptor LYVE-1 as described previously by using overnight incubation with goat anti-mouse CD31 FITC (1:100; Santa Cruz Biotechnology, Dallas, TX, USA) and goat anti-mouse LYVE-1 (1:400; AF2125, R&D Systems, MN, USA)^41,54^. A Cy3-conjugated donkey anti-goat antibody (1:2,000, Jackson ImmunoResearch Laboratories, West Grove, PA, USA) was then added as the secondary antibody and incubated for another 2 h. Stained whole mount corneas were mounted in Vectashield with 4,6-diamidino-2-phenylindole (Vector Laboratories Inc., Burlingame, CA, USA). Images of stained whole mount corneas were taken under a fluorescence microscope (BZ-X71000, KEYENCE, Osaka, Japan). The fractions of total corneal areas covered by blood and lymphatic vessels were then calculated by using ImageJ (National Institutes of Health, Bethesda, MD, USA; available at http://rsb.info.nih.gov/ij/index.html)^41,55^.

### Statistical analysis

Experiments with more than two groups were analyzed by using one-way or two-way ANOVA, followed by the post hoc Bonferroni’s multiple comparison test if F value indicated a significant main factor effect or interaction between factors. All statistical calculations were performed using Prism 9.1.0 (216) software (GraphPad, La Jolla, CA, USA). Data are presented as the mean ± standard error of mean (SEM). The differences were considered statistically significant if *P* < 0.05. A heatmap was created by using Prism heatmap function to illustrate the distribution of fold changes in mRNA expression levels for several genes encoding angiogenic factors.

## Data Availability

All datasets generated during or analyzed during this study are included in this published article.
